# The Administration of Gas and Oxygen

**Published:** 1920-04-17

**Authors:** 


### THE NEW ANÆSTHETIC.

### The Administration of Gas and Oxygen.

During the war the administration of nitrous oxide gas with oxygen had a tremendous vogue in the surgery of the casualty clearing-stations and the base hospitals. So popular was this method of anæsthesia, that it came to be regarded as the ideal anæsthetic, which in due time would almost entirely displace other modes of anæsthesia. We doubt, however, if an anæsthetic which will fulfil all demands will ever be discovered, so great are the varieties of the surgery of this epoch. Spinal anæsthesia, the last great anæsthetic discovery, was heralded in much the same way, but in due course was found to be extremely useful for many purposes, but not for all.

### Administration and Apparatus.

The pioneer apparatus of the late Sir Frederick Hewitt is less convenient than the newer models on the sight-feed principle, invented by an American, Dr. Gwathmey. A really satisfactory apparatus is that of Dr. Geoffrey Marshall, or the more portable apparatus manufactured by Mayer and Phelps to the design of Dr. F. E. Shipway is equally useful.

The great advantages of these types of apparatus are:—

1. Ease of manipulation;

2. A fairly exact index of the proportion of the gases administered;

3. A graduated degree of re-breathing is permissible, with a corresponding deepening of the anæsthesia;

4. The anæsthetic may be "reinforced" with ether vapour.

Briefly stated, the apparatus is designed to allow nitrous oxide and oxygen to bubble separately through a modified Wolff's bottle, and then to pass directly to the face-piece, or, if desired, to bubble a second time through a bottle containing anæsthetic ether. The face-piece, which is of the familiar Hewitt's "dental type," is connected with a small rubber bag, a valve intervening, so that a certain amount of rebreathing may be allowed.

Induction should never be hurried; two or three minutes should be given to secure a tranquil anæsthesia. Once fully anæsthetised the colour of the patient must be carefully watched, and if the slightest tinge of cyanosis be present more oxygen should be supplied; this manœuvre may lighten the anæsthesia, but in spite of this disadvantage the course we have indicated is the safe and proper one to follow.

### The Advantages of Gas and Oxygen.

From the patient's point of view, which after all is the most important, there is the advantage of a speedy and tranquil induction of anæsthesia followed at the end of the operation by a speedy recovery to consciousness and a refreshing absence of the post-operative nausea usually experienced after ether or chloroform. Moreover, nourishment may be taken as soon as the patient desires it, unless the operation be an abdominal one. This state of affairs contrasts very favourably with the long and tedious induction with ether or chloroform, and the long, dazed "recovery" from such anæsthetics, accompanied as they often are by sickness, which in the case of abdominal wounds makes the patient extremely uncomfortable, and predisposes towards the formation of an "incisional hernia."

Another great advantage of gas and oxygen is the comparative freedom from post-anæsthetic bronchitis or pneumonia which has been observed after this mode of anæsthesia. Again, in those conditions which contra-indicate chloroform or ether, we may with advantage use this anæsthetic. To give a few examples, chloroform is contra-indicated in operations for eclampsia and acute suppurative conditions, whilst gas and oxygen produce no harmful effects. Ether is contra-indicated in inflammatory states of the chest, such as bronchitis or pneumonia, also in cases of nephritis and high blood pressure from any cause; in all these operative complications the new anæsthetic may be used with advantage.

During the war this anæsthetic was used with great success in cases of "shock," and where the patient was ex-sanguine from loss of blood: in civil practice this knowledge has been applied in its use for grave cases of ruptured abdominal viscera, such as the spleen or an ectopic gestation.

### Disadvantages.

The gravest disadvantage, in our opinion, is the lack of "depth" in the anæsthesia produced; as a rule, a flaccid condition of the muscles is required in abdominal operations and this result cannot always be obtained. This raises the question which is exercising the minds of most anæsthetists at the present time. Should a general anæsthetic ever be pushed so far as to secure muscular relaxation? There are many who believe it is preferable, whenever possible, to secure relaxation by local analgesia or anæsthesia.

For these reasons gas and oxygen has recently been used in conjunction with a spinal anæsthetic, the former to secure a decerebrate condition of the patient, and the latter to secure a perfect muscular relaxation and a condition of anoci-association by which the patient is spared much shock. Such a combined anæsthetic is the nearest approach to the ideal as yet, the surgeon having a maximum convenience in his work afforded by the flaccid and "still" abdomen, and the patient all the advantages peculiar to gas and oxygen.

### Is Gas and Oxygen a Safe Anæsthetic?

The early results of the new anæsthetic pointed to a lower mortality than is usual with ether or chloroform, and it is with great disappointment that we have to record from later statistics mortality equal to, though no greater than, that due to ether. The Americans were the first to describe sudden syncope associated with the new anæsthetic; without any warning the patient's heart would stop

**The New Anæsthetic**—(*continued*).

beating, then respiration fail, and in spite of every endeavour to resuscitate the patient, the syncope would terminate in death.

A number of such casualties have been described during the past few years, and pathologists as yet cannot state the cause with any certainty. At *post-mortem* examinations the heart in nearly every case has been found to be acutely dilated, and this has presumably been the cause of death. In the particular types of apparatus at present used, the anæsthetist must realise that the *only oxygen given to the patient is through the apparatus*, in other words the apparatus is a "closed" one: thus the physiologists incline to the belief that the tissues, and more particularly nerves, such as the vagus and phrenic, are in these fatal cases, gradually starved of the oxygen necessary to complete their metabolism. The obvious answer to this state of affairs is to give more oxygen, and the anæsthetist must resolutely refuse to push his patient into a deeper anæsthesia at the expense of *safety*.

Conclusions.

We consider gas and oxygen a most valuable addition to the present choice of anæsthetics, but it should be reserved for those cases where no great degree of muscular relaxation is sought. This, we think, should be its routine employment in healthy cases: but, in addition, it has its uses in those conditions where a "general anæsthetic" is contra-indicated, or where the state of the patient is very anxious from "shock" or loss of blood. Combined with spinal anæsthesia the new anæsthetic provides anæsthesia almost ideal for pelvic operation and others which require a maximum of muscular relaxation and a minimum of shock.

At the present time, continuous gas and oxygen is chiefly employed in hospital practice, its use in private being much curtailed by the number, weight, and expense of the cylinders of gas required.

This anæsthetic should be administered only by those experienced in the special administration of anæsthetics. If this principle were adhered to the present mortality would tend to diminish.

